# Supplementary material for: The Evolution and Characterization of the RNA Interference Pathways in Lophotrochozoa
Source: Genome Biol Evol. 2024 May 7;16(5):evae098. doi: 10.1093/gbe/evae098 (PMC11114477; doi:10.1093/gbe/evae098)
Supplement: evae098_Supplementary_Data [file evae098_supplementary_data.zip › Supplementary_materials.zip!/selected_miRNAs/NoGe_selected_miRNAs/mirdeep_pdf_files/NMRB01003740.1_Notospermus_geniculatus_strain_Ushimado_scaffold3740,_whole_genome_shotgun_sequence_44407.pdf]

Provisional ID	:	NMRB01003740.1_Notospermus_geniculatus_strain_Ushimado_scaffold3740,_whole_genome_shotgun_sequence_44407
Score total	:	155551.3
Score for star read(s)	:	3.9
Score for read counts	:	155542.7
Score for mfe	:	1.7
Score for randfold	:	0
Score for cons. seed	:	3
Total read count	:	305102
Mature read count	:	303575
Loop read count	:	0
Star read count	:	1527

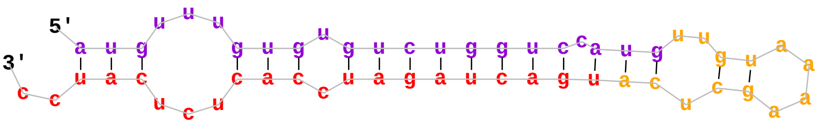

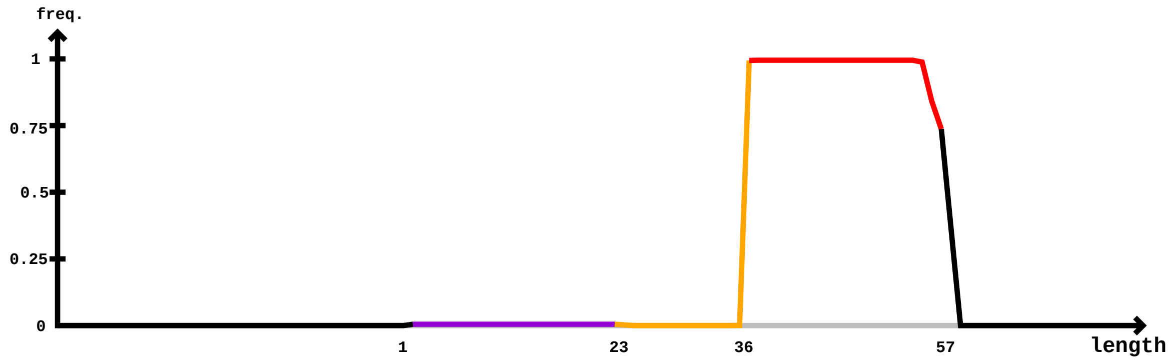

Star **Mature**

```
5'- ggcauucaacaugggcaguugugccagucuuuaggauguuugugugucugguccauguuguaaaagcucaugacuagauccacucucauccugacggcuuggaaucaucua  -3'   obs
    ggcauucaacaugggcaguugugccagucuuuaggauguuugugugucugguccauguuguaaaagcucaugacuagauccacucucauccugacggcuuggaaucaucua         exp
    ((((..((((.......))))))))((((.((((((((...(((.((((((((.(((..((....)).)))))))))))).)))...)))))))).))))............   reads   mm   sample
    ...................................auguuugugugucuggucca........................................................   9       0    Fm3
    ...................................auguuugugugucugguGca........................................................   1       1    Fm3
    ...................................auguuugugugucugguccau.......................................................   6       0    Fm3
    ...................................auguuugugugucugguccauA......................................................   1       1    Fm3
    ...................................auguuugugugucugguGcaug......................................................   1       1    Fm3
    ...................................auguuugugugucugguccaug......................................................   555     0    Fm3
    ...................................auguuugugugucugAuccaug......................................................   1       1    Fm3
    ...................................auguuugugugucuggCccaug......................................................   3       1    Fm3
    ...................................auguuuguUugucugguccaug......................................................   1       1    Fm3
    ...................................auguuugugugucuggAccaug......................................................   1       1    Fm3
    ...................................auguuuguguUucugguccaug......................................................   1       1    Fm3
    ...................................auguuugugugucugguAcaug......................................................   7       1    Fm3
    ...................................auguuuguguAucugguccaug......................................................   4       1    Fm3
    ...................................auguuugugugucuggGccaug......................................................   2       1    Fm3
    ...................................auguuugugugucugguUcaug......................................................   4       1    Fm3
    ...................................auguuugugugucugguccaugu.....................................................   1       0    Fm3
    ...................................auguuugugugucugguccaugA.....................................................   2       1    Fm3
    ...................................auguuugugugucugguccauguu....................................................   2       0    Fm3
    ...................................auguuugugugucugguccauguug...................................................   2       0    Fm3
    ...................................auguuugugugucugguccauguugu..................................................   1       0    Fm3
    ....................................uguuugugugucugguccaug......................................................   10      0    Fm3
    ....................................uguuugugugucuCguccaug......................................................   6       1    Fm3
    ....................................uguuugugugCcugguccaug......................................................   1       1    Fm3
    ....................................uguuugugugucuggucUaug......................................................   1       1    Fm3
    ....................................uguuugugugucugguccaugu.....................................................   2       0    Fm3
    .....................................................................augacuagauccacAcucau......................   26      1    Fm3
    .....................................................................augacuagauccacAcucauc.....................   4       1    Fm3
    .....................................................................augacuagauccacAcucaucc....................   40      1    Fm3
    ......................................................................ugacuagauccacAcuca.......................   583     1    Fm3
    ......................................................................ugacuagauccacCcucau......................   4       1    Fm3
    ......................................................................ugacuagauccacucuAau......................   1       1    Fm3
    ......................................................................ugacuagauccacucucau......................   10      0    Fm3
    ......................................................................ugacuagauccacAcucau......................   38247   1    Fm3
```

```
                                   Star                                 Mature
ggcauucaacaugggcaguugugccagucuuuaggauguuugugugucugguccauguuguaaaagcucaugacuagauccacucucauccugacggcuuggaaucaucua
......................................................................ugacuagauccacGcucau......................    9       1    Fm3
......................................................................ugacuagauccacucucauc.....................    2       0    Fm3
......................................................................ugacuagauccacAcucauc.....................    7777    1    Fm3
......................................................................ugacuagauccacGcucaucc....................    15      1    Fm3
......................................................................ugacuagauccacucuGaucc....................    1       1    Fm3
......................................................................ugacuagauccacucuUaucc....................    1       1    Fm3
......................................................................ugacuagauccacucucaucc....................    19      0    Fm3
......................................................................ugacuagauccacCcucaucc....................    3       1    Fm3
......................................................................uCacuagauccacucucaucc....................    1       1    Fm3
......................................................................ugacuagauccacAcucaucc....................    109454  1    Fm3
......................................................................ugacuagauccacGcucauccu...................    1       1    Fm3
......................................................................ugacuagauccacucucauccA...................    1       1    Fm3
......................................................................ugacuagauccacucucauccu...................    2       0    Fm3
......................................................................ugacuagauccacAcucauccu...................    6735    1    Fm3
......................................................................ugacuagauccacAcucauccug..................    5       1    Fm3
.......................................................................gacuagauccacAcucau......................    38      1    Fm3
.......................................................................gacuagauccacAcucauc.....................    6       1    Fm3
.......................................................................gacuagauccacAcucaucc....................    118     1    Fm3
.......................................................................gacuagauccacucucaucc....................    1       0    Fm3
.......................................................................gacuagauccacAcucauccu...................    6       1    Fm3
........................................................................acuagauccacAcucaucc....................    5       1    Fm3

...................................auguuugugugucugguGc.........................................................    1       1    Mm3
...................................auguuugugugucugguccaug......................................................    61      0    Mm3
...................................auguuugugugucugguAcaug......................................................    1       1    Mm3
...................................auguuugugugucugguGcaug......................................................    1       1    Mm3
...................................auguuugugugucugguccaugu.....................................................    2       0    Mm3
...................................auguuugugugucugguccauguugu..................................................    1       0    Mm3
.....................................................................augacuagauccacAcucauc.....................    2       1    Mm3
.....................................................................augacuagauccacAcucaucc....................    8       1    Mm3
......................................................................ugacuagauccacAcuca.......................    30      1    Mm3
......................................................................ugacuagauccacCcucau......................    1       1    Mm3
......................................................................ugacuagauccacAcucau......................    235     1    Mm3
......................................................................ugacuagauccacGcucauc.....................    2       1    Mm3
......................................................................ugacuagauccacAcucauc.....................    3336    1    Mm3
......................................................................ugacuagauccacucucauc.....................    3       0    Mm3
......................................................................ugacuagaGccacucucaucc....................    1       1    Mm3
......................................................................ugacuagauccacGcucaucc....................    6       1    Mm3
......................................................................ugacuagauccacAcucaucc....................    31930   1    Mm3
......................................................................ugacuagauccacucucaucc....................    8       0    Mm3
......................................................................ugacuagauccacAcucauccu...................    2807    1    Mm3
.......................................................................gacuagauccacAcucau......................    1       1    Mm3
.......................................................................gacuagauccacAcucauc.....................    6       1    Mm3
.......................................................................gacuagauccacAcucaucc....................    35      1    Mm3
.......................................................................gacuagauccacAcucauccu...................    5       1    Mm3
.........................................................................cuagauccacAcucaucc....................    1       1    Mm3

...................................auguuugugugucugguc..........................................................    1       0    Fm1
...................................auguuugugugucuggCccaug......................................................    1       1    Fm1
...................................auguuugugugucugguGcaug......................................................    1       1    Fm1
...................................auguuugugugucugguccaGg......................................................    1       1    Fm1
...................................auguuugugugucugguUcaug......................................................    1       1    Fm1
...................................auAuuugugugucugguccaug......................................................    1       1    Fm1
...................................auguuugugugucuggucUaug......................................................    1       1    Fm1
...................................auguuugugugucugguccaug......................................................    219     0    Fm1
...................................auguuugugugucugguAcaug......................................................    2       1    Fm1
...................................auguuugugugucuggGccaug......................................................    2       1    Fm1
...................................auguuugugugucugguccaugA.....................................................    1       1    Fm1
...................................auguuugugugucugguccauguuguaGaagcuca.........................................    3       1    Fm1
.....................................................................augacuagauccacAcucauc.....................    10      1    Fm1
.....................................................................augacuagauccacAcucaucc....................    3       1    Fm1
......................................................................ugacuagauccacAcuca.......................    104     1    Fm1
......................................................................ugacuagauccacAcucau......................    1663    1    Fm1
......................................................................ugacuagauccacAcucauc.....................    2197    1    Fm1
......................................................................ugacuagauccacCcucaucc....................    1       1    Fm1
......................................................................ugacuagauccacAcucaucc....................    13070   1    Fm1
......................................................................ugacuagauccacGcucaucc....................    2       1    Fm1
......................................................................ugacuagauccacucucaucc....................    2       0    Fm1
......................................................................ugacuagauccacAcucauccu...................    613     1    Fm1
......................................................................ugacuagauccacAcucauccug..................    1       1    Fm1
```

Star **Mature**

ggcauucaacaugggcaguugugccagucuuuaggauguuugugugucugguccauguuguaaaagcucaugacuagauccacucucauccugacggcuuggaaucaucua			
....gacuagauccacAcucauc.....................	1	1	Fm1
....gacuagauccacAcucaucc....................	5	1	Fm1
....gacuagauccacAcucauccu...................	1	1	Fm1
...................................auguuuguguAucugguccaug..	1	1	Fm2
...................................auguuugugugucugguccaug..	403	0	Fm2
...................................auguuugugugucuggCccaug..	1	1	Fm2
...................................auguuugugugucugguAcaug..	1	1	Fm2
...................................aCguuugugugucugguccaug..	1	1	Fm2
...................................auguuugugugucuggGccaug..	1	1	Fm2
...................................auguuugugugucugguccaGg..	4	1	Fm2
...................................auguuugugugucuggucUaug..	1	1	Fm2
...................................auguuugugugucugguUcaug..	2	1	Fm2
...................................auguuuguguCucugguccaug..	1	1	Fm2
...................................Uuguuugugugucugguccaug..	2	1	Fm2
...................................auguuugCgugucugguccaug..	1	1	Fm2
...................................auguuugugugucugguGcaug..	2	1	Fm2
...................................auguuugugugucugguccaugA...	1	1	Fm2
....ugacuagauccacAcuca.......................	30	1	Fm2
....ugacuagauccacAcucau......................	312	1	Fm2
....ugacuagauccacAcucauc.....................	4299	1	Fm2
....ugacuagauccacCcucaucc....................	1	1	Fm2
....ugacuagauccacGcucaucc....................	4	1	Fm2
....ugacuagauccacucucaucc....................	3	0	Fm2
....ugacuagauccacAcucaucc....................	17744	1	Fm2
....ugacuagauccacAcucauccu...................	681	1	Fm2
....ugacuagauccacAcucauccug..................	5	1	Fm2
....gacuagauccacAcucaucc....................	4	1	Fm2
....acuagauccacAcucauc.....................	1	1	Fm2
...................................auguuuguguCucugguccaug..	1	1	Mm1
...................................Guguuugugugucugguccaug..	1	1	Mm1
...................................auguuugugugucuggAccaug..	1	1	Mm1
...................................auguuugugugucugguccaug..	134	0	Mm1
...................................auguuugugugucugguUcaug..	1	1	Mm1
...................................auguuugugugucugguAcaug..	1	1	Mm1
...................................auguuugugugucugguccauguuguaaaagcuca...	1	0	Mm1
....augacuagauccacAcucauc.....................	20	1	Mm1
....augacuagauccacAcucaucc....................	10	1	Mm1
....ugacuagauccacAcuca.......................	1309	1	Mm1
....ugacuagauccacAcucau......................	1692	1	Mm1
....ugacuagauccacAcucauc.....................	13643	1	Mm1
....ugacuagauccacGcucauc.....................	2	1	Mm1
....ugacuagauccacCcucauc.....................	3	1	Mm1
....ugacuagauccacucucauc.....................	4	0	Mm1
....ugacuagauccacAcucaucc....................	26840	1	Mm1
....ugacuagauccacGcucaucc....................	2	1	Mm1
....ugacuagauccacucucaucc....................	4	0	Mm1
....ugacuagauccacAcucauccu...................	1161	1	Mm1
....gacuagauccacAcucauc.....................	14	1	Mm1
....gacuagauccacAcucaucc....................	14	1	Mm1
...................................auguuugugugucugguc..	1	0	Mm2
...................................auguuugugugucugguccaug..	40	0	Mm2
...................................Uuguuugugugucugguccaug..	1	1	Mm2
...................................auguuugugugucugguccaugu...	1	0	Mm2
....augacuagauccacAcucau......................	1	1	Mm2
....augacuagauccacAcucaucc....................	6	1	Mm2
....ugacuagauccacAcuca.......................	48	1	Mm2
....ugacuagauccacAcucau......................	2319	1	Mm2
....ugacuagauccacGcucau......................	1	1	Mm2
....ugacuagauccacAcucauc.....................	765	1	Mm2
....ugacuagauccacucucaucc....................	2	0	Mm2
....ugacuagauccacAcucaucc....................	12824	1	Mm2
....ugacuagauccacGcucauccu...................	1	1	Mm2
....ugacuagauccacAcucauccu...................	591	1	Mm2
....ugacuagauccacAcucauccug..................	1	1	Mm2
....gacuagauccacAcucaucc....................	9	1	Mm2
